# Composting of Olive Mill Pomace, Agro-Industrial Sewage Sludge and Other Residues: Process Monitoring and Agronomic Use of the Resulting Composts

**DOI:** 10.3390/foods10092143

**Published:** 2021-09-10

**Authors:** Alessandro Leone, Roberto Romaniello, Antonia Tamborrino, Luciano Beneduce, Anna Gagliardi, Marcella Giuliani, Giuseppe Gatta

**Affiliations:** 1 Department of Agricultural and Environmental Science (DiSAAT), University of Bari Aldo Moro, Via Amendola 165/A, 70126 Bari, Italy; alessandro.leone@uniba.it; 2 Department of Agriculture, Food, Natural Resources and Engineering (DAFNE), University of Foggia, Via Napoli 25, 71122 Foggia, Italy; roberto.romaniello@unifg.it (R.R.); luciano.beneduce@unifg.it (L.B.); anna.gagliardi@unifg.it (A.G.); marcella.giuliani@unifg.it (M.G.); giuseppe.gatta@unifg.it (G.G.)

**Keywords:** co-composting, sewage sludge, olive mill wastes, composting plant, processing tomato, durum wheat

## Abstract

The viability of co-composting of olive mill pomace added to sewage sludge with other organic residues was evaluated and the agronomic use of the final composts was investigated. Two composting piles at different carbon-nitrogen ratios were performed, in which olive mill pomace (OMP), sewage sludge from vegetable processing (SS), fresh residues from artichoke processing residues (AR), and wheat straw (WS) were used. The two composting piles were placed inside a specially built greenhouse and a turning machine pulled by a tractor was used for turning and shredding the organic matrix (every 6 days) during the process. The humidity and temperature of organic matrices have been monitored and controlled during the entire composting process, which lasted 90 days. The process was also monitored to evaluate the microbiological safety of the final compost. The humidity of both piles was always kept just above 50% until the end of the thermophilic phase and the maximum temperature was about 50 °C during the thermophilic phase. The carbon-nitrogen ratio decreased from 21.4 and 28.2, respectively (initial value at day 1 in Pile A and B), to values ranging from 12.9 to 15.1, both composts that originated from the two different piles were microbiologically safe. During a two-year period, the effects of different types of compost on the main qualitative parameters of processing tomato and durum wheat was evaluated. Five fertilization treatments were evaluated for tomato and durum wheat crops: unfertilized control (TR_1_); compost A (TR_2_); compost B (TR_3_); ½ mineral and ½ compost A (TR_4_); and mineral fertilizer commonly used for the two crops (TR_5_). Concerning the processing tomato yield, TR_5_ and TR_4_ showed the best results (2.73 and 2.51 kg, respectively). The same trend was observed considering the marketable yield per plant. The only difference was related to the treatments that included the compost (2.32, 1.77, and 1.73 kg/plant for TR_4_, TR_3_, and TR_2_, respectively). As regards the qualitative parameters of tomato, the highest average weight of the fruits was found in the TR_5_, TR_4_, and TR_3_ treatments (respectively, 73.67 g, 70.34 g, and 68.10 g). For durum wheat, only the protein component was differentiated between treatments. Furthermore, wheat grain yield parameters generally increased by combined application of mineral fertilizer and compost.

## 1. Introduction

Aerobic composting is a biochemical process of organic matter degradation in hu-mic compounds that are more stable [1,2,3]. It is based on the action of aerobic microorganisms; therefore, the process must take place under good ventilation conditions and an appropriate oxygen concentration. The final compost is then usually used for agronomic purposes to improve plant growth [4,5].

Composting is a simple method to use, has few process requirements, has relatively low investment costs, and can be conveniently carried out using organic waste matrices from different agro-food chains.

It is also fundamental to correctly manage the process to improve the reproduction conditions of microorganisms, in order to reduce the total process time until a mature compost suitable for agricultural purposes is obtained. In order for the microorganisms to multiply rapidly and carry out the degradation of organic matter, humidity and temperature of the composting matrix must be properly checked and adjusted throughout the process, in addition to an appropriate carbon-nitrogen ratio (C/N) of the initial matrix in the input of the process.

In the countries of the Mediterranean basin, an abundant by-product that often does not have adequate reuse is olive mill pomace. According to the estimates of the International Olive Council (IOC), based on the data of the 2020/2021 crop year, Mediterranean countries account for approximately 98% of the global olive oil production. The most common technologies for olive oil extraction involve the continuous extraction system with a decanter centrifuge in a two- and three-phase configuration. However, between the two configurations, the former is by far the most used. Two-phase processing produces wet pomace with 65–70% of moisture [6].

Due to the high water mass fraction of the wet husk, it is not convenient to use it for energy purposes or to extract the oil chemically; therefore, it is often disposed of on the ground (in accordance with local regulations), thus increasing the olive oil production cost. The disposal of olive mill waste is a critical environmental problem for Mediterranean countries [7].

Composting appears to be one of the most promising options to transform these residues into a valuable organic soil conditioner. Most research on composting processing of olive mill by-products used pomace from a two-phase mill combined with other by-products, with the aim to increase the porosity of the initial matrix and providing an appropriate C/N ratio (range: 20–40) for the good operation of the process [8].

Previous studies used different types of by-products to increase the matrix porosity of the composting process, such as olive leaves and cereal straw [9], pine sawdust [10], wheat straw [11], wool waste and wheat straw [12], and wood chips and rice by-products [4]. The other by-products used to reach an appropriate C/N ratio include chicken manure [9,13] and rabbit and sheep manure [14,15]. In other research, sewage sludge was added to the pomace olive in order to regulate the carbon-nitrogen ratio [16,17,18].

Worldwide, every year, the treatment of water produces millions of tons of residual sludge. The sludge generated by the wastewater purification processes is destined to different fates: it can be incinerated through the combustion process, accumulated in specific landfills, composted, recycled as building materials, or used in agriculture as a soil improver [19,20].

The effects of the use in agriculture as soil improvers of sludge derived from water purification processes have been evaluated in numerous studies. These have shown how the administration of sludge can improve the chemical, physical, and biological properties of the soil [21,22] and can positively affect the production of many crops through a significant increase in nutrients and organic matter [23,24,25,26,27].

Nevertheless, sewage sludge, especially if it comes from domestic or industrial waste, contains pathogens, heavy metals, and organic toxic pollutants that limit its land application [28,29]. This waste must be properly managed to prevent environmental problems and risks for human health [30]. This aspect has led, in recent years, to a contraction in the agricultural use of civil and industrial sludge due to fears relating to the accumulation of toxic substances in soils and crops. Therefore, when sludge is included in the composting mix, microbial monitoring is essential to avoid potential hazards and to deliver a final product that complies with local and national regulations [31].

It should be emphasized that, among the different sewage sludges, those produced from wastewater purification processes of the agro-food industries (e.g., companies processing fruit and vegetables) have very low or no heavy metal content and a higher organic substance content than the sludge coming from the purification of civil wastewater; therefore, they are more suitable for agricultural use.

Taking into account the importance of valorizing organic waste from the food industry, minimizing the environmental impact of the sewage sludge, and, finally, the need to return mature organic substance to the soil as a soil improver for crops, this paper aimed to evaluate the technical feasibility of co-composting of four types of organic wastes, namely olive mill pomace (OMP), sewage sludge from vegetable processing (SS), fresh residues from artichoke processing residues (AR), and wheat straw (WS). To this purpose, a composting plant equipped with a machine for turning and shredding the windrow was used. Finally, the agronomic use of the two different composts produced at the end of the composting process was investigated.

## 2. Materials and Methods

### 2.1. Experimental Design

In the Cannone Industrie Alimentari company (Cerignola—FG), by using an experimental greenhouse, two composting experiments were initiated using partially pitted olive mill pomace (OMP), sewage sludge from vegetable processing (SS), fresh residues from artichoke processing residues (AR), and wheat straw (WS).

The addition of wheat straw has been necessary to improve the granulometric structure and avoid the compaction of the mixture during the composting phase.

Raw materials were placed into a covered rectangular windrow 1.3 wide and 0.7 m high inside the greenhouse. The composting process lasted 90 days, the last 30 of which were for the maturation process. The 90-day period was chosen in accordance with [12,32], who used olive mill pomace as a base matrix in the composting process.

During the first 60 days, the pile was turned every 6 days by using the compost turning machine described below. Periodic humidity adjustment with the addition of tap water was made every 6 days to keep moisture levels high (above 50%). The humidity correction was carried out for the first 60 days of the process. 

Both the turning of the windrows and the humidity regulation were not carried out during the compost maturation phase.

Continuous measurement of the temperature and humidity values by means of a set of datalogger was carried out for each pile.

### 2.2. Greenhouse Design for Composting Operations

**Figure 1:**
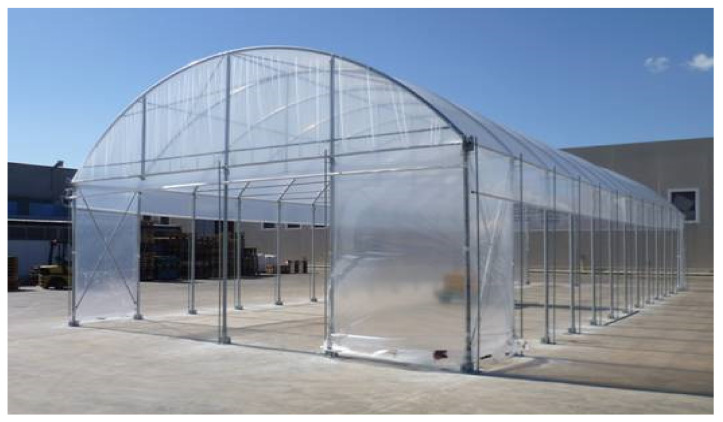
Greenhouse structure built for the composting tests.

An arched greenhouse with a curved roof and pillars (Figure 1) was designed and built for composting tests. The pillars, like the entire structure, are made up of metal profiles, metal boxes, to resist the atmospheric agents and atmospheres that are created inside the greenhouses, according to the characteristics of the UNI EN 13031 standard. The roof consists of a steel structure 8.0 m wide and 19.5 m long covered with a transparent polyvinylchloride (PVC) film, with a usable surface area of 156 m^2^. The greenhouse was solidly anchored to the reinforced and waterproofed floor, equipped with channels for the recovery of liquid leaks.

Two 4-m wide openings have been provided on the two heads to allow access to the vehicles for unloading the initial matrices, loading the compost at the end of the process, and access to the tractor with the turner machine to carry out the periodical reshuffling of the matrices. Inside the plant, two 2-m wide side lanes have been provided for the arrangement of two swaths during the composting phase at a distance of 0.5 m from the sidewall of the greenhouse. Between the two composting lanes, a 3-m central lane has been planned for the passage of vehicles and operators. The main geometric data are shown in Table 1.

### 2.3. Compost Turning Machine

**Figure 2:**
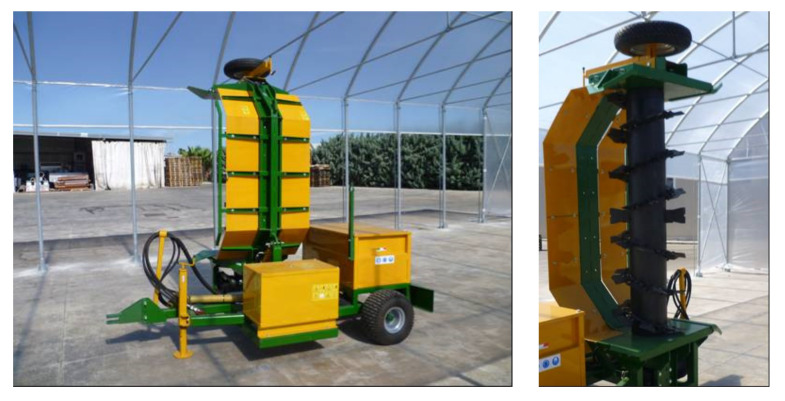
Turner machine (**left**); detail of the rotating shaft (**right**).

The machine used for turning and shredding the organic matrix is pulled by a tractor (Figure 2).

This machine has a rear member consisting of a tunnel-shaped metal casing, carrying a horizontal reel inside with two sets of counter-rotating vanes. The rotating reel is moved by means of a cardan shaft connected to the Power Take-Off (P.T.O.) of the tractor.

The main components of the machine are the following:

- Main bearing frame, made of painted steel, with a drawbar for towing and a hinge for connecting the turning element, supported by two bearing wheels;
- System for the hydraulic adjustment of the free height to the ground;
- Counterweight on the bearing frame;
- Turning device with a length of 1.4 m and a height of 0.8 m, equipped with turning hammers welded to the reel, having the shape of a double opposed helicoid;
- Structure supporting the turning mechanism with a hinge for connection to the mainframe;
- Metal roof and load-bearing lintel;
- Double-acting hydraulic cylinder for overturning the turning element;
- Gearbox for the deviation of the motion to the turning organ;
- Cardan shaft for the connection between the P.T.O. tractor side and the P.T.O. operator side;
- Complete hydraulic system for operating the lifting cylinder of the turning element;
- System for the sprinkling of liquids with an external water connection.

The purpose of the machine is to break up the formed aggregates, aerate the material, and finally homogenize the pile, resulting in better uniformity and ensuring optimal conditions of temperature and oxygen.

The machine is also equipped with a water sprinkling system, supplied by water mains, on the mass during turning, to regulate the optimal humidity conditions. The liquid sprinkling system is completed with a flow adjustment valve and nozzles arranged along the entire profile of the crankcase.

During use, the turning section is arranged laterally to the tractor, allowing the latter to travel parallel to the pile. In addition, in transport conditions, the turning section can be folded into a vertical position, allowing the machine to reenter the tractor track.

### 2.4. Sampling and Analytical Methods

For the residues used in the experiment and for composting mixture on the first, sixtieth, and ninetieth days of the composting process, ten subsamples of composting material were taken at random points of each pile and carefully mixed to form a composite sample.

Moisture content (drying at 105 °C) and pH (1:25 water extract) were determined in the samples according to standard procedures.

Total organic carbon (TOC) and total nitrogen (TN) were determined on a dried sample using an elemental analyzer (CE Instruments, CHNS-O Model EA-111) after treatment in 1 M HCl to remove carbonates.

The humic fraction was analyzed as described by [33]. Carbon of humic acid (HA) and fulvic acid (FA) were analyzed using the CE Instrument (model NCS 2500) and Shimadzu Total Organic Carbon Analyzer TOC-500, respectively.

Macro-micronutrients (sodium, potassium, magnesium, calcium, and nitrogen (N-NO_3_ and N-NH_4_)) content was determined by ion-exchange chromatography (Dionex ICS-1100, Dionex Corporation, Sunnyvale, CA, USA).

N-NO_3_ content was extracted from samples (0.5 g DW) with 50 mL 3.5 mmol L^−1^ Na_2_CO_3_ and 1.0 mmol L^−1^ NaHCO_3_, and it was separated and quantified using Dionex equipped with an IonPac AG14 precolumn and an IonPac AS14 column.

Sodium, potassium, magnesium, calcium, and N-NH_4_ contents were analyzed after incineration of samples (1.0 g DW) at 550 °C and acid digestion in 20 mL of 1.0 mol L^−1^ HCl at 99.5 ± 0.5 °C, for 30 min. The resulting solution was filtered, diluted, and quantified using Dionex equipped with an IonPac CG12A guard column and an IonPac CS12A analytical column. The data were expressed as mg kg^−1^ dry weight (DW).

The available phosphorus content was determined by the Olsen method.

For analytical determination of the total heavy metal content in the composts (Cd, Cr, Ni, Pb, Cu, and Zn), the samples (about 0.5 g dw) were mineralized in 10 mL HNO_3_/H_2_O_2_ (3:1; *v*/*v*) in a microwave oven (CEM-Mars6). After cooling, the digested samples were diluted with Milli-Q water to 50 mL [34] and analyzed by ICP-OES (Agilent, ICP-OES 720).

### 2.5. Composting Process

Table 2 shows the analytical characteristics of the residues used in the experiment. Two piles (A and B) that differ in the C/N ratio were prepared using the matrices previously described. The matrices were arranged in layers within the covered structure on the concrete slab. The mixing of the matrices was carried out by turning the layers several times, until its complete homogenization, using a turning machine. Water was added to the mass thus prepared until optimal humidity was reached. The percentage compositions of the piles are based on the analytical characteristics of the initial matrices (Table 3 and Table 4).

The two piles differed from each other due to the different C/N ratios, i.e., 21.4 in pile A and 28.2 in pile B. Three temperature sensors (Delta Ohm HD2301.0) were placed in the core of the windrow to measure the thermal profile of the composting mixture during the process. The sensors were removed before the mechanical turning and shredding operation. In the full thermophilic phase, at day 52, to highlight the thermal profile of the two windrows, thermal images of the mounds and inside their sections were acquired by means of a thermal camera (Flir E40bx).

Moisture evaluation of the composting mixture was carried out every 4 days using a thermobalance (OHAUS MB45) on a mixture sample. The moisture measurement operation was repeated five times.

**Figure 3:**
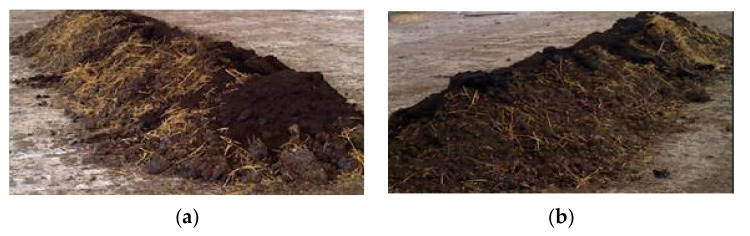
Pile A (**a**) and B (**b**) at day 0.

Initial piles are shown in Figure 3a,b.

At the end of the composting process, Compost A was produced from pile A and Compost B from pile B. The compost was then used for agronomic tests.

### 2.6. Microbiological Analyses

Total bacteria, fecal coliforms, *Escherichia coli*, and fecal enterococci were enumerated by plate count methods as reported in previous research work [35], while detection of *Salmonella* spp. was conducted according to Italian guidelines for the microbial analyses of compost [36]. Quantitative Real-time PCR (qPCR) was used as a sensitivity method to detect pathogenic target microorganisms. The presence of the human pathogens *Listeria monocytogenes*, *Salmonella* spp., and *E. coli* O157:H7 was investigated, by using methods previously reported [37].

### 2.7. Qualitative and Quantitative Parameters of the Tomato and Durum Wheat Crops

**Figure 4:**
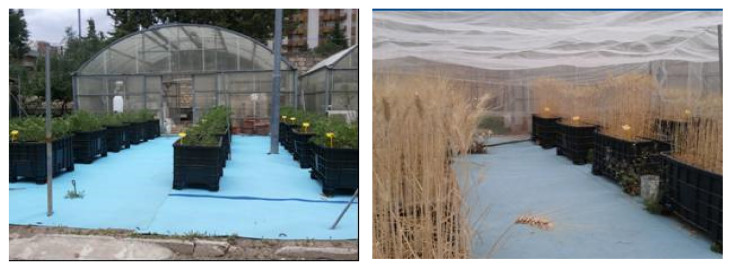
Experimental tests relating to the cultivation of industrial tomatoes and durum wheat.

During a two-year period, an evaluation was carried out of the effects of different types of compost on the main qualitative parameters of processing tomato (cv Ulisse—S&G Syngenta Seeds S.p.A.) and durum wheat crops (cv Saragolla S&G Syngenta Seeds S.p.A.) cropped into polyethylene containers (length, 1.0 m.; width, 0.63 m.; height, 0.64 m) (Figure 4). The experimentation was carried out at the Campus of the Department of Agricultural Sciences, Food, Natural Resources and Engineering (DAFNE) of the University of Foggia (15°31′53″ E; 41°27′23″ N). Both crops were grown on a clay soil (United States Department of Agriculture Classification, Washington, DC, USA) with the following main chemical characteristics: organic matter (Walkley–Black method), 0.9%; available phosphorus (Olsen method), 22.0 mg kg^−1^; exchangeable potassium (ammonium acetate method), 470 mg kg^−1^; total nitrogen (Dumas method), 1.1‰; ammonium-nitrogen (N-NH_4_) and nitrate-nitrogen (N-NO_3_), 6.1 mg/kg and 3.1 mg/kg, respectively.

Five fertilization management strategies (treatments) were evaluated for tomato and durum wheat crops (Table 5): (i) unfertilized control (TR_1_); (ii) compost A (TR_2_); (iii) compost B (TR_3_); (iv) ½ mineral and ½ compost A (TR_4_); (v) mineral fertilizer commonly used for the two crops (TR_5_). The five experimental treatments were arranged by a randomized block experimental design with three replicates.

After the harvest, the marketable and discarded fruit were counted and weighted to estimate the total yield (kg/plant) and marketable yield (kg/plant). Moreover, on a sample of 10 marketable fruit from each experimental treatment (TR_1_-TR_5_), the following qualitative parameters were measured: mean weight, the soluble solids content of the flesh (°Brix), pH of tomato juice, titratable acidity (g citric acid 100 mL^−1^ fresh juice) [38], and dry matter content (%) [39]. External fruit color was measured using a colorimeter CM-700d spectrophotometer (Minolta Camera Co. Ltd., Osaka, Japan), with a D65 light source based on the CIELAB color space represented by L* a* b* values. Measurements were taken at four randomly selected areas of the fruit surface and mean values were used for analyses of the a*/b* ratio (color index; CI), which represents an index that sufficiently describes the color changes of tomato fruit [40].

Regarding the durum wheat crop, the number of spiklets/ears, the number of kernels for each ear, thousand kernel weight, plant height, and ear length were determined at the harvest. Protein, calcium, potassium, magnesium, and sodium content of the kernels were determined. Protein content was then calculated by multiplying the total nitrogen (Kjeldahl method) by the coefficient 6.25. The sodium, potassium, magnesium, and calcium content in wheat grain was determined by ion-exchange chromatography (Dionex ICS-1100, Dionex Corporation, Sunnyvale, CA, USA). Anions were extracted from samples (0.5 g DW) with 50 mL 3.5 mmol L^−1^ Na_2_CO_3_ and 1.0 mmol L^−1^ NaHCO_3_, and they were separated and quantified using Dionex equipped with an IonPac AG14 precolumn and an IonPac AS14 column. Cations were analyzed after incineration of samples (1.0 g DW) at 550 °C and acid digestion in 20 mL of 1.0 mol L^−1^ HCl at 99.5 ± 0.5 °C, for 30 min. The resulting solution was filtered, diluted, and quantified using Dionex equipped with an IonPac CG12A guard column and an IonPac CS12A analytical column. The data were expressed as mg kg^−1^ dry weight (DW) [41].

### 2.8. Statistical Analysis

The datasets were tested according to the basic assumptions of analysis of variance (ANOVA). The normal distribution of the experimental error and the common variance of the experimental error were verified through Shapiro–Wilk and Bartlett’s tests, respectively. The differences in the means were determined using Tukey’s honest significance difference post-hoc tests at the 5% probability level.

ANOVA analysis was performed using the JMP software package, version 14.3 (SASInstitute Inc., Cary, NC, USA).

## 3. Results and Discussion

### 3.1. Temperature and Moisture Trends

**Figure 5:**
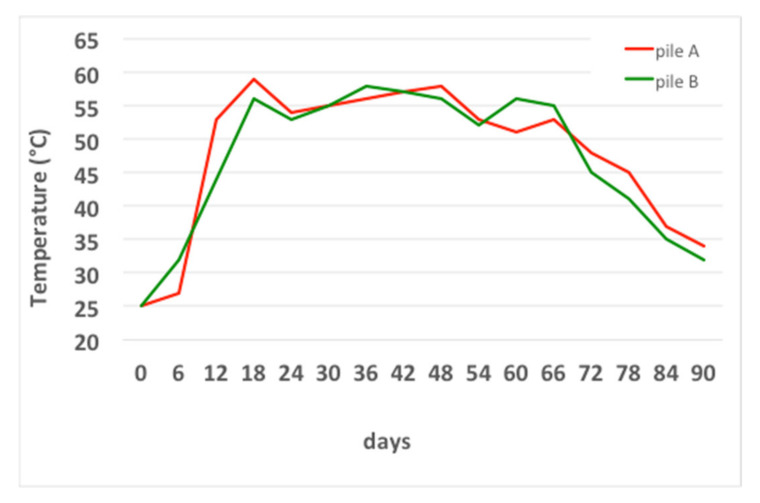
Temperature temporal variation in the compost windrows.

The evolution of the temperature is shown in Figure 5. The temperature of the piles reached 50 °C after about 12 days of the first mesophilic phase, probably because of the fast depletion of oxygen, as reported in [14]. Subsequently, the temperature remained constant, with values between 50 °C and 55 °C, for about 54 days, characterizing the thermophilic phase. After 66 days of the process, the temperature gradually decreased to 32–34 °C on the ninetieth day, with about 24 days of the second mesophilic phase and maturation of the compost.

The same temperature trend was also observed by [42] in a composting process using olive leaves and pomace from a three-phase olive mill plant. The temperature remained at about 45 °C for about 40 days and then decreased. A similar trend was observed in [43].

**Figure 6:**
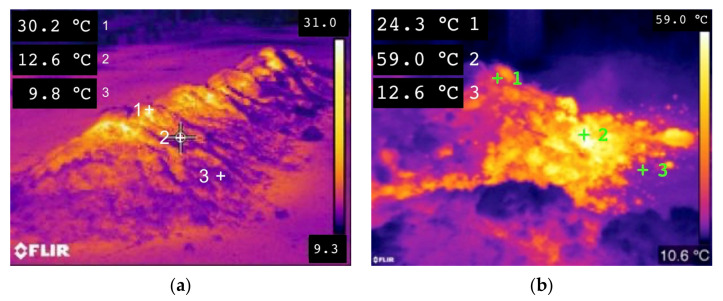
Thermal images acquired at day 40. Outside temperature profile (**a**); inside temperature profile (**b**).

In the full thermophilic phase, at day 40, to highlight the thermal profile of the two windrows, thermal images of the mounds and inside their sections were acquired by means of a thermal camera (Flir E40bx) (Figure 6).

The thermographic images show what happens during the process on the pile before being subjected to turning, during the composting phase. As shown in Figure 6a,b, the part of the pile at the lowest temperature is the portion located at the base, due to its thin layer and the large contact surface with the floor. In the upper part of the pile, on the other hand, higher temperatures were noted probably because of rising heat towards the top of the pile. Figure 6a shows how, after the pile disruption, the inner portion of the pile has a great thermal content (59 °C), able to transfer the heat to the upper part of the pile. The more the inner temperature is high, the more the heat transfer from the core to the top of the pile is enhanced, in particular in the thermophilic phase.

This shows that in the full thermophilic phase, there is a thermal difference of over 25 °C between the outside and the core of the product. A similar thermal difference is highlighted in [42], where the authors assert that from days 7 and 17, compost temperature was found to be higher in the center of the windrow and lower at the edges, reporting as a possible cause the influence of air temperature.

**Figure 7:**
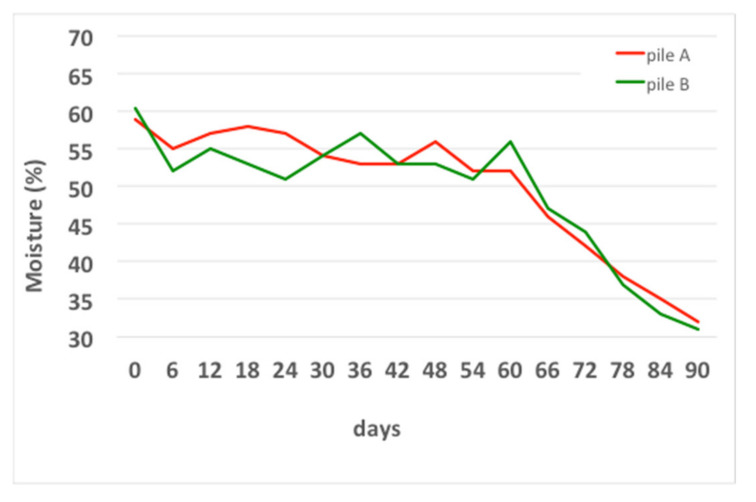
Moisture temporal variation in the compost windrows.

The evident thermal non-uniformity underlines the non-uniformity of the process. This experimental evidence confirms the need to carry out mixing and turning operations to uniform the mass and to give all the particles the possibility of occupying different positions in the pile throughout the process, to standardize the biological degradation between the particles. In Figure 7, the moisture temporal variation in the compost windrows is shown.

The graph shows the trend of the moisture measured using thermobalance after the addition of tap water to adjust the humidity above 50%, as reported in [42].

The graph shows how the humidity value was correctly adjusted up to the sixtieth day of the process, after which no more water was added, so that the compost maturation phase could be carried out correctly.

The composting process was completed on the ninetieth day and the two compost A and B were produced from piles A and B.

Table 6 shows the physicochemical characteristics of the compost material (Pile A and B) from the various composting stages (i.e., day 1, day 60, and day 90).

On the first day, pH was 6.9 in Pile A and 6.6 in Pile B, while at the end of the composting process, pH was 7.7 and 7.3, respectively, which is the result of compost maturation under aerobic conditions. On day 60, pH increased to 7.9–8.2, probably due to the biodegradation of acids containing carboxylic and phenolic groups and the mineralization of organic compounds, as reported in [43,44].

The C/N ratio decreased from 21.4 and 28.2 (initial values at day 1) to values ranging from 12.9 to 15.1, which are more appropriate for agricultural use, and similar to that obtained by [14,45]. A significant carbon depletion was obtained in both experiments. Considering the optimal temperature trend, it was supposed that a significant part of the carbon was removed by aerobic degradation, confirming that both the artichoke residues and the straw used provided good porosity to the matrix to obtain a good air circulation; moreover, the turning operation was effective.

The humic acid fraction is an indicator of the organic material transformation during the composting process. In both windrows, HA + FA increased with compost stability. Three months after starting, the HA + FA content was 20.4% in Pile A and 24.1% in Pile B.

Regarding the macro and micronutrient content (Table 6), the nitrogen content (N-NO_3_ and N-NH_4_) was higher in Pile A than in Pile B (189.2 vs. 168.0 mg/kg dw and 50.0 vs. 39.2 mg/kg dw for N-NO_3_ and N-NH_4_, respectively). The same behavior was observed for phosphorus and potassium (301 mg/kg dw vs. 189 mg/kg dw and 1214.9 g/100 g dw vs. 816.4 g/100 g dw for phosphorus and potassium, respectively).

The concentration of heavy metals in the sewage sludge can be very high [46]; therefore, it is very important to assess the content of these elements in compost derived by composting of sewage sludge. Under our experimental conditions, the content of heavy metals (Ni, Pb, Cu, and Zn) was also higher in Pile A than in Pile B, while the content of Cd and Cr was below the detection limit. In both piles, the examined heavy metals were lower than the threshold values listed in the national and international guidelines for the different matrices (soil and vegetables) [46].

The results obtained are confirmed by different studies in which the composting of olive mill pomace added to sewage sludge was carried out [16,17,47].

### 3.2. Microbial Quality of the Produced Compost

**Figure 8:**
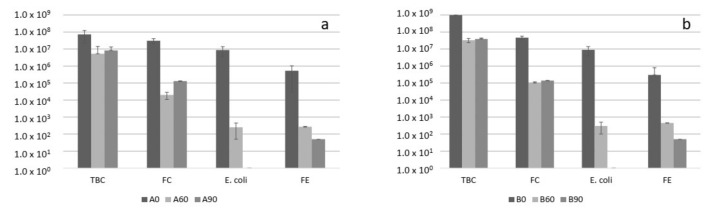
Microbiological indicator values of compost pile A (**a**) and B (**b**) during the composting process. Data are expressed in CFU/g. TBC = total bacterial count, FC = fecal coliforms, FE = fecal enterococci.

As evidenced in Figure 8, both compost piles had similar starting quantities of the main microbiological indicators, including fecal indicators (coliforms, enterococci, and *E. coli*). During the first phase (Day 60) and maturation of the compost (Day 90), all fecal indicators dropped to a very low level. In both composts, the *E. coli*, considered the main indicator of compost microbial quality and safety, was not detectable with culture methods. At the same time, *Salmonella* spp. was not detected by culture methods independently from compost pile and time of sampling. The data confirmed that co-composting WWTP sludge causes an unavoidable increase of *E. coli* and other fecal indicators level, but if thermophilic phase reaches sufficient time/temperature levels, *E. coli* die-off is sufficient to fulfill US-EPA and EU regulations [48].

Table 7 resumes the result obtained by qPCR in order to detect with high sensitivity, potential contamination of the compost piles by human pathogens. Interestingly, both the composting piles were found to be positive for *Salmonella* and *Listeria monocytogenes* at the beginning of the process. However, at the end of the thermophilic phase (day 60) and after maturation, no pathogen was detected in any of the samples analyzed. Our results shows that the contamination of raw feedstock by human pathogens must be taken into account, particularly when dealing with sludge [49]. However, process conditions (duration and temperature of the thermophilic phase), together with ammonia release and competition with competitive compost microflora, could be the main factor that led to the inactivation of pathogens in our trial [50]. Regarding microbial safety, the proposed process allowed to deliver a final compost suitable for agronomic application onto soil.

### 3.3. Agronomics Results

Concerning the processing tomato yield (Table 8), TR_5_ and TR_4_ (mineral fertilization and compost A + mineral fertilizer, respectively) showed the best results. The total yields for each plant were 2.73 and 2.51 kg for TR_5_ and TR_4_, respectively. The other treatments (TR_3_, TR_2_, and TR_1_) determined lower yields not statistically different from each other. The same trend was observed considering the marketable yield per plant. The only difference was related to the treatments that included the compost (TR_4_, TR_3_, and TR_2_). In this case, the marketable yield resulted to be not statistically different between TR_4_ and the other treatments, including compost. The marketable yield resulted in 2.32, 1.77, and 1.74 kg/plant for TR_4_, TR_3_, and TR_2_, respectively. The different production behavior between TR_2_ and TR_3_ vs. TR_4_ and TR_5_ is probably due to the slow degradation process of the organic compound contained in the compost and, consequently, to the low nutrients releasing [51].

The qualitative parameters of tomato are shown in Table 9. The data show how the main qualitative characteristics influenced by the different treatments compared were all those referable to the morphometric parameters of the fruit (average weight and equatorial and longitudinal diameter of the fruits), while the average values of the other parameters (dry matter, pH, soluble solids content, and titratable acidity) are not statistically significantly different. The highest average weight of the fruits was found in the TR_5_, TR_4,_ and TR_3_ treatments (respectively, 73.67 g, 70.34 g, and 68.10 g). The TR_1_ treatment (unfertilized control) was the one that showed the lowest values of equatorial/longitudinal diameter and color index.

Table 10 shows the values of the morpho-productive parameters measured at the end of the wheat crop cycle. Except for the thousand kernel weight, all parameters showed a better behavior of the TR_5_ treatment, which was not statistically different from TR_4_.

Table 11 highlights the main qualitative parameters of wheat grain for the different treatments compared. Differently from morpho-productive parameters, only the protein component was differentiated between treatments, while the other parameters (the content of calcium, potassium, magnesium, and sodium) do not seem to be affected. In particular, the treatment in which the traditional dose of mineral fertilization (TR_5_) was supplied and the TR_4_ treatment, in which the compost was also associated with mineral fertilization, resulted in higher protein content values (about 12%) compared to the other treatments. This protein content is the minimum level required for the pasta supply chain.

These different results concerning grain protein content could be due to the higher nitrogen mineral level (N-NH_4_ and N-NO_3_) supplied to the wheat by T_4_ and T_5_ with respect to other treatments. Many studies have suggested that high doses of nitrogen tend to increase the amount of grain protein content [52,53,54]

Therefore, the combined use of compost and mineral fertilization (TR_4_) could be a possible solution in the fertilization of durum wheat in some agronomic contexts. In particular, TR_4_ could be applied in areas where it is very important to contain the mineral fertilizer inputs (e.g., integrated production systems of durum wheat) or on soils where the organic matter content is very low.

In accordance with similar studies [55,56,57,58,59], our results indicated that wheat grain yield parameters generally increased by combined application of mineral fertilizer and compost. These effects are probably related to a positive effect of organic matter on soil structure, which led to better root development and, consequently, to a more nutrient uptake [51,54].

## 4. Conclusions

According to the estimates of the Population Division of the United Nations Department of Economic and Social Affairs, in 2019, the world population is 7.7 billion people, which could grow to around 8.5 billion in 2030, 9.7 billion in 2050, and 10.9 billion in 2100. This global trend towards a larger world population will lead to a substantial increase in global demand for food, which will strongly impact the agro-industrial sector. The consequent need to produce more food will lead to the production of high amounts of organic solid wastes, wastewater, and sewage sludge, whose proper management will be necessary to avoid environmental problems. Consequently, the need to preserve agroecosystems must be paired with a proper management of crop fertilization.

This study has shown that it is possible to carry out a composting process of different organic matrices from different food chains carefully mixed together by adequately controlling/adjusting the process parameters, until a stable compost for agronomic purposes is obtained.

The results of our study showed that a part the required fertilizing elements of the tomato and wheat crops could be provided by compost and, thus, alleviate the environmental hazards on agroecosystems (e.g., fertilizer leaching).

## Figures and Tables

**Table 1:** Dimensional parameters of the greenhouse.

Parameter	Value (m)
Eaves Height	2.50
Ridge height	4.50
Distance between the pillars	1.50
Width	8.00
Length	19.50

**Table 2:** Analytical and calculated characteristics of the residues used in the experiment. Olive mill pomace, partially destoned (OMP), sewage sludge (SS), artichoke residues (AR), and wheat straw (WS).

Analytical and Calculated Characteristics	OMP	SS	AR	WS
MOISTURE %	65.0	84.0	82.1	7.0
TOTAL ORGANIC CARBON (TOC) (%)	47.2	27.1	2.2	44.0
TOTAL NITROGEN (TN) (%)	0.87	4.7	40.4	0.5
C/N RATIO	54.25	5.7	19.4	88.0

**Table 3:** Composition of the piles used in the experiment in percentages.

Matrix	Pile A	Pile B
OLIVE MILL POMACE (OMP)	50.0%	60.0%
SEWAGE SLUDGE (SS)	40.0%	25.0%
ARTICHOKE RESIDUES (AR)	8.5%	13.5%
WHEAT STRAW (WS)	1.5%	1.5%
TOTAL WEIGHT OF THE PILE (KG)	4400	4200

**Table 4:** Analytical and calculated characteristics of the initial composting mixtures.

Analytical and Calculated Characteristics	Pile A	Pile B
PH (1:25)	6.9	6.6
MOISTURE %	58.8	60.4
C/N RATIO	21.4	28.2
HA + FA	14.3	16.1

**Table 5:** Fertilization treatments applied to tomato and durum wheat crops.

TR_1_	TR_2_	TR_3_	TR_4_	TR_5_
processing tomato				
Control (unfertilized)	Compost A 1.5 kg/m^2^	Compost B 1.5 kg/m^2^	Compost A (0.75 kg/m^2^) + Mineral fertilization (8.0 g N/m^2^; 16.0 g P_2_O_5_/m^2^)	Mineral fertilization (16.0 g N/m^2^; 16.0 g P_2_O_5_/m^2^)
durum wheat				
Control (unfertilized)	Compost A (0.78 kg/m^2^)	Compost B (1.25 kg/m^2^)	Compost A (0.38 kg/m^2^) + Mineral fertilization (5.3 g N/m^2^; 4.6 g P_2_O_5_/m^2^)	Mineral fertilization (10.6 g N/m^2^; 9.2 g P_2_O_5_/m^2^)

TR_1_ = unfertilized; TR_2_ = compost A; TR_3_ = compost B; TR_4_ = ½ mineral and ½ compost A TR_4_; TR_5_ = conventional mineral fertilizer.

**Table 6:** Analytical and calculated characteristics of the composting mixtures.

Parameter	Pile A			Pile B		
	Day 1	Day 60	Day 90	Day 1	Day 60	Day 90
*Physical parameters*						
pH (1:25)	6.9	8.2	7.7	6.6	7.9	7.3
Temperature (°C)	25.1	56.5	34.2	25.3	58.7	32.1
Moisture (%, *w*/*w*)	58.8	53.7	32.4	60.4	57.2	31.6
C/N ratio (-)	21.4	18.5	12.9	28.2	21.6	15.1
HA + FA (% of organic matter)	14.3	19.2	20.4	16.1	23.2	24.1
*Macro-micronutrients* ^†^						
P_2_O_5_ (mg/kg dw) ^††^			301			189
N-NO_3_ (mg/kg dw)			189.2			168.0
N-NH_4_ (mg/kg dw)			50.0			39.2
N total (g/100 g dw)			2.4			2.1
K (g/100 g dw)			1214.9			816.4
Ca (g/100 g dw)			1609.3			1980.6
Mg (g/100 g dw)			357.5			334.5
Na (g/100 g dw)			1990.7			1405.8
*Heavy metal* ^†^						
Cd (g/100 g dw)			nd			nd
Cr (g/100 g dw)			nd			nd
Ni (g/100 g dw)			3.5			1.8
Pb (g/100 g dw)			7.2			3.3
Cu (g/100 g dw)			41.5			20.1
Zn (g/100 g dw)			80.3			60.4

^†^ These parameters were only evaluated at the end of the composting stages (day 90). ^††^ (determined by the Olsen method). nd, not detected, DW, dry weight.

**Table 7:** Real-time quantitative PCR detection of human pathogens during the composting process. Positive PCR reactions are reported as “+” when triplicate samples from the same composting piles were analyzed and at least one replicate was positive. Negative reactions are reported as “-” (all replicates are negative).

Compost Pile	Day 0		Day 60		Day 90	
	A	B	A	B	A	B
*Salmonella* spp.	+	+	-	-	-	-
*Listeria monocytogenes*	+	+	-	-	-	-
*E. coli* O157:H7	-	-	-	-	-	-

**Table 8:** Effect of fertilization management strategies on the main quantitative parameters of the processing of tomato crop.

Parameter	Fertilization Treatment				
	TR_1_	TR_2_	TR_3_	TR_4_	TR_5_
Total yield (kg/plant)	1.71 b	1.89 b	1.88 b	2.51 a	2.73 a
Marketable yield (kg/plant)	1.45 c	1.74 bc	1.77 bc	2.32 ab	2.48 a
Discarded yield (kg/plant)	0.25 a	0.15 bc	0.11 c	0.19 abc	0.21 ab

TR_1_ = Unfertilized control; TR_2_ = compost A; TR_3_ = compost B; TR_4_ = ½ mineral and ½ compost A fertilization; TR_5_ = mineral fertilizer. In each row, values followed by different letters are significantly different at *p* ≤ 0.05 according to Tukey’s test.

**Table 9:** Effect of fertilization management strategies on the main qualitative parameters of the processing tomato fruits.

Production Parameter	Compared Theses				
	TR_1_	TR_2_	TR_3_	TR_4_	TR_5_
Dry matter (% fw) ^†^	5.78 a	5.51 a	5.81 a	5.97 a	6.00 a
Mean weight (g)	64.20 b	64.66 b	68.10 ab	70.34 ab	73.67 a
Polar diameter (mm)	61.00 b	65.00 b	67.33 ab	69.32 ab	70.10 a
Equatorial diameter (mm)	30.76 c	33.00 bc	35.66 abc	39.00 ab	42.00 a
pH (-)	4.20 a	4.07 a	4.40 a	4.36 a	4.60 a
Soluble Solid Content (°Brix)	4.78 a	4.89 a	4.96 a	5.02 a	5.10 a
Titratable acidity (g citric acid 100 mL^−1^ fresh juice)	0.30 a	0.28 a	0.32 a	0.31 a	0.33 a
Color index (a/b) ^††^	0.90 b	1.08 a	1.02 a	1.15 a	1.17 a

^†^ fw, Fresh weight, ^††^ a, red/green chromaticity (negative values indicate green, while positive values indicate red); b, yellow/blue chromaticity (negative values indicate blue, while positive values indicate yellow). Thus, the higher the a/b ratio, the higher the red color of tomato fruit. TR_1_ = unfertilized control; TR_2_ = compost A; TR_3_ = compost B; TR_4_ = ½ mineral and ½ compost A fertilization; TR_5_ = mineral fertilizer. In each row, values followed by different letters are significantly different at *p* ≤ 0.05 according to Tukey’s test.

**Table 10:** Morpho-qualitative parameters of wheat crop.

Treatment	Plant Height (cm)	Ear Length (cm)	Spiklet (n)	Kernel/Ear (n)	Thousand Kernel Weight (g)
TR_1_	50.8 b	5.2 b	11.0 b	16.6 b	43.9 a
TR_2_	57.6 ab	6.1 b	11.6 b	23.6 ab	46.7 a
TR_3_	53.8 b	6.2 b	12.3 b	23.7 ab	50.9 a
TR_4_	63.4 ab	6.5 ab	12.3 b	24.3 ab	43.5 a
TR_5_	69.2 a	7.6 a	16.0 a	31.3 a	44.5 a

TR_1_ = unfertilized control; TR_2_ = compost A; TR_3_ = compost B; TR_4_ = ½ mineral and ½ compost A fertilization; TR_5_ = mineral fertilizer. In each column, values followed by different letters are significantly different at *p* ≤ 0.05 according to Tukey’s test.

**Table 11:** Qualitative parameters wheat grain yield.

Treatment	Proteins	Calcium	Potassium	Magnesium	Sodium
	% dw ^†^	[mg/kg fw ^††^]	[mg/kg fw ^††^]	[mg/kg fw ^††^]	[mg/kg fw ^††^]
TR_1_	9.1 c	453.5 a	3651.2 a	589.9 a	134.6 a
TR_2_	9.7 b	419.7 a	4077.2 a	610.1 a	193.7 a
TR_3_	10.2 b	403.1 a	3323.7 a	597.7 a	156.7 a
TR_4_	11.9 ab	347.7 a	3259.0 a	654.3 a	152.8 a
TR_5_	12.1 a	664.6 a	3946.1 a	724.0 a	169.8 a

^†^ dw, dry weight; ^††^ fw, fresh weight.TR_1_ = Unfertilized control; TR_2_ = compost A; TR_3_ = compost B; TR4 = ½ mineral and ½ compost A fertilization; TR5 = mineral fertilizer. Different letters in columns denote statistically significant differences at *p* < 0.05 (Tukey’s test).

## Data Availability

Data not available.
